# Forest aboveground biomass estimation using Landsat 8 and Sentinel-1A data with machine learning algorithms

**DOI:** 10.1038/s41598-020-67024-3

**Published:** 2020-06-19

**Authors:** Yingchang Li, Mingyang Li, Chao Li, Zhenzhen Liu

**Affiliations:** 1 Co-Innovation Center for Sustainable Forestry in Southern China, College of Forestry, Nanjing Forestry University, Nanjing, 210037 China; 2 College of Forestry, Shanxi Agricultural University, Jinzhong, 030801 China

**Keywords:** Ecological modelling, Forest ecology, Forestry

## Abstract

Forest aboveground biomass (AGB) plays an important role in the study of the carbon cycle and climate change in the global terrestrial ecosystem. AGB estimation based on remote sensing is an effective method for regional scale. In this study, Landsat 8 Operational Land Imager and Sentinel-1A data and China’s National Forest Continuous Inventory data in combination with three algorithms, either the linear regression (LR), random forest (RF), or the extreme gradient boosting (XGBoost), were used to estimate biomass of the subtropical forests in Hunan Province, China. XGBoost is a scalable tree boosting system that is widely used by data scientists and provides state-of-the-art results for many problems. It can process an entire dataset with billions of examples using a minimal amount of computational resources through the particular way of cache access patterns, data compression, and data fragmentation. The results include: (1) The combination of Landsat 8 and Sentinel-1A images as predictor variables in the XGBoost model provided the best AGB estimation. (2) In contrast to the LR method, the F-test results indicated that a significant improvement in AGB estimation was achieved with the RF and XGBoost algorithms. (3) The effect of parameter optimization was found to be more significant on XGBoost than on RF. (4) The XGBoost model is an effective method for AGB estimation and can reduce the problems of overestimation and underestimation. This research provides a new way of estimating AGB for the subtropical forest based on remote sensing through the synergy of different sensors datasets and modeling algorithms.

## Introduction

The forest ecosystem is the largest and most important natural ecosystem in the terrestrial ecosystem, in which it plays a crucial role in maintaining global ecological balance as well as promoting global biological evolution and community succession^1,2^. Forest biomass is an important variable for evaluating carbon sequestration and carbon balance capacity of forest ecosystems. Accurate estimation of forest biomass is particularly important for studying the carbon cycle of the terrestrial ecosystem in large areas^3,4^.

Forest aboveground biomass (AGB) is mainly estimated by traditional field measurements^5^ or remote sensing methods^6^. For a small forest stand, the AGB calculation is more accurate when based on actual field measurements. However, the use of field measurements to calculate forest AGB is not feasible at the regional scale, as it is too costly, labor intensive, and time consuming. Previous studies have shown that remote sensing can effectively measure and monitor forest biomass at the regional scale; therefore, various types of remote sensors, that use both passive and active sensors, have been used to estimate AGB^7–9^. As an active type of remote sensing, radar has the ability to penetrate the canopy and interact with the main biomass components, i.e., the tree trunks and branches. The radar backscattering intensity increases as forest biomass increases. Radar has different sensitivity to forest biomass according to its wavelength. As the wavelength increases, the scattering saturation value increases, and the correlation between radar backscatter and biomass also increases^10–12^; thus, long wavelength bands are more suitable for biomass estimation. However, using longer wavelength radar data is not always feasible because most longer wavelength satellites are commercial satellites and their data is very expensive. The European Space Agency’s Sentinel-1 mission with C-band has provided high spatial resolution synthetic aperture radar (SAR) data freely for all users worldwide. However, these data alone are not sufficient to estimate forest biomass due to the low penetration and saturation point of the C-band. In addition to SAR data, the optical remotely-sensed data, such as Landsat, SPOT, WorldView-2, Sentinel-2, and their products, such as vegetation indices and texture images, have also been shown to be closely related to biomass^13,14^. Previous studies have shown that synergistic use of data from different sources to estimate biomass is more accurate than data from a single source^15,16^, especially in the tropical and subtropical regions where the stand structure and tree species are complex^3,17^. To date, the performance of the combination of Landsat 8, which is NASA’s eighth satellite in the Landsat series (the most widely used optical satellites) and continues the former mission to provide world land cover change information, and Sentinel-1, which is operated by the European Space Agency and is the first of the five missions of earth observation satellites as part of the Global Monitoring for Environment and Security, in AGB modeling has not been sufficiently studied.

In addition, it is critical to select an appropriate algorithm to establish AGB estimation models. The classical statistical regression method is simple and easy to calculate. The linear regression (LR) method was the most widely used method for AGB estimation in the previous studies^6,18^. A statistical regression model can be built using the sample data and remote sensing parameters. However, the classical statistical regression method does not effectively describe the complex nonlinear relationship between forest AGB and remote sensing data. To improve the nonlinear estimation ability of the biomass model, machine learning methods, such as decision tree, K-nearest neighbor (KNN), artificial neural network (ANN), and support vector machine (SVM), are applied to the remote sensing estimation of the forest AGB^19,20^. Previous studies have indicated that algorithms based on the decision tree, such as random forest (RF) and gradient boosting (GB), have an excellent performance in biomass estimation modeling^21,22^. Moreover, there are many adjustable parameters of the machine learning algorithms, which are very important to the models but the tuning processes were overlooked sometimes. Previous studies have shown that the sensibility of the parameters of RF and stochastic gradient boosting were different, and the tuning processes was significantly important to the performance of the models^23,24^.

In this study, the AGB of a subtropical forest has been estimated using Landsat 8 optical datasets in combination with Sentinel-1 SAR datasets, and the LR and two machine learning algorithms—RF and extreme gradient boosting (XGBoost), as an advanced GB system, is widely used by data scientists and provided state-of-the-art results for many problems—were used to predict AGB. Despite the excellent performance of XGBoost, XGBoost has many parameters that need to be tuned. To date, few researchers have used XGBoost to estimate forest aboveground biomass based on remote-sensing data. Li *et al*. used data from two sources, China’s National Forest Continuous Inventory and Landsat 8, in combination with three separate algorithms, LR, RF, and XGBoost, to establish biomass estimation models^25^. The results indicated that the XGBoost models significantly improved the estimation accuracy compared with the LR models, and reduced the problems of overestimation and underestimation to a certain extent; however, the tuning process was not explained in detail. The specific objectives of this study were as follows: (1) to explore the process and effect of tuning parameters for the RF and XGBoost; (2) to validate the ability of the RF and XGBoost for estimating AGB; (3) to compare the accuracy of the LR, RF, and XGBoost models using different datasets; and (4) to draw the AGB map for the study area.

## Materials and methods

### Study area

**Figure 1:**
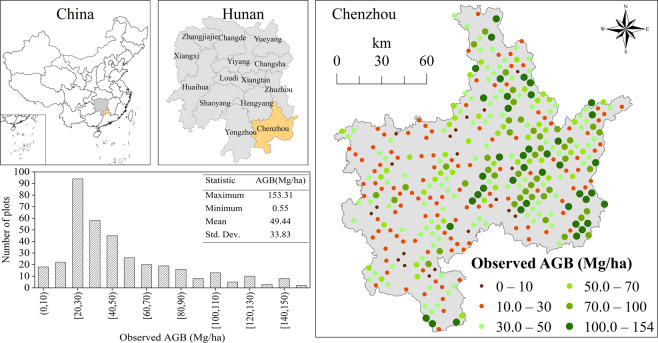
The location of the study area, including the distribution histogram of the observed AGB values of the field plots.

Chenzhou City (19.39 × 10^3^ km^2^, 24°53′N–26°50′N, 112°13′E–114°14′E) is situated in southeastern Hunan Province, China (Fig. 1). Most of the study areas are located in a subtropical monsoon humid climate zone and the regional natural conditions, such as vegetation, are greatly affected by the southern mountains. There are many types of forest vegetation, mainly subtropical evergreen; therefore, there is no significant difference in forest canopy cover in one year. The forest area is 12.08 × 10^3^ km^2^, accounting for 62.30% of the study area.

### Inventory data

The eighth data (in 2014) of China’s National Forest Continuous Inventory (NFCI), which is the first level of forest inventory system of China, were used in this study (Fig. 1). The size of each plot is 25.82 m × 25.82 m (approximately 0.0667 ha). The AGB of a tree was calculated by using the general one-variable (i.e., the diameter at breast height) individual tree aboveground biomass model based on wood density for 34 tree species in China^26^. The plot AGB was converted to per hectare biomass (Mg/ha). Note that the plots that were situated on non-forestry land, such as farmland, water area, and construction land, were eliminated. Finally, 367 plots, which recorded 26,387 trees, were used in this study (Fig. 1). In general, the average AGB was 49.44 Mg/ha within the range of 0.55 to 153.31 Mg/ha and a standard deviation (SD) of 33.83 Mg/ha.

### Satellite data

#### Data processing

**Figure 2:**
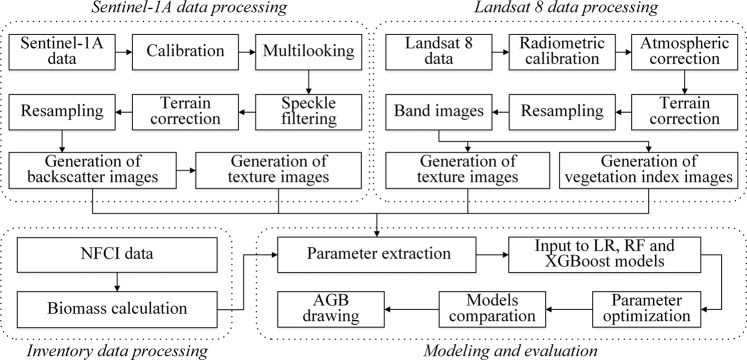
Workflow of the AGB estimation model.

The SAR data used in this study were acquired from the Sentinel-1A satellite, which is a C-band SAR with a central frequency of 5.405 GHz^27^. In this study, two Ground Range Detected (GRD) images, which are the level 1 products of Sentinel-1A with two polarization images, are used. The Sentinel-1A GRD images were acquired in October of 2015 (Supplementary Table S1). Sentinel-1A GRD data was processed by the Sentinel Application Platform. In additional, Landsat 8 Operational Land Imager (OLI) satellite images were also used in this study. The Landsat 8 images were acquired in October of 2015 (Supplementary Table S1). The images of the study area were nearly cloud-free. Landsat 8 OLI data were processed by the Environment for Visualizing Images software. The workflow of data processing used in this study is shown in Fig. 2. The images were resampled to a pixel size of 25.82 m same as the inventory plot. The texture images were calculated using grey-level co-occurrence matrix algorithm with a 5 × 5-pixel window^28^. Landsat 8 OLI and Sentinel-1A GRD data were processed by the Environment for Visualizing Images software (Version 5.3.1, Boulder, Colorado, USA) and Sentinel Application Platform software (Version 6.0.6, Paris, France), respectively.

#### Extraction of remote sensor parameters from a field plot

The center coordinates of each plot were generated based on the southwest vertex coordinates of the plot. The plot boundary does not completely coincide with the pixel of the remote sensing image, although the images were resampled to the same size as the field plots. The first step was to create a 25.82 m radius buffer around each plot center to minimize the effect of these location offsets. The buffer radius was limited at 25.82 m to prevent intrusion to the surrounding area where the tree canopy cover is different. Then, the mean pixel value, which was used as the plot value, was extracted for the buffer area of each plot center.

Finally, the remote sensing predictor variables included the primal images of 6 Landsat band images and 2 SAR backscatter images as well as the generated images of 6 vegetation index images, 48 Landsat texture images, and 16 SAR texture images (Supplementary Table S2). In this study, Landsat and Sentinel images were used respectively as predictor variables to build the models, and then, a combination of two datasets were used as predictor variables.

### Models

#### LR

The LR assumes a linear relationship between a response and a set of explanatory variables, that is, AGB and remote sensing predictor variables. In this study, stepwise linear regression was used to establish a relationship between the observed AGB and the predictor variables with the Landsat 8, Sentinel-1A, and the combined dataset. Stepwise regression was performed in SPSS software (Version 25, Armonk, New York, USA), the confidence level was set at 95%, and the probability of the F-test was set to 0.05 and 0.10 for entry and removal, respectively. The multicollinearity was evaluated for the selected predictor variables using the variance inflation factors.

#### RF

In the RF algorithm, a bootstrap sample of the training data is chosen, namely, 90% of the training dataset is chosen randomly to build a decision tree, and the remaining part of the training dataset is used for estimating out-of-bag (OOB) error for each tree. At each node of the tree, a small sample of explanatory variables is chosen randomly to determine the best split. RF has been applied extensively as a classification algorithm^29^ and has been used for time series forecasting in large-scale regression-based applications^30,31^.

#### XGBoost

XGBoost was proposed by Chen *et al*.^32^ who had obtained state-of-the-art performance in Kaggle machine learning competitions. Compared with the general gradient boosting, XGBoost performs a second-order Taylor expansion for the objective function and uses the second derivative to accelerate the convergence speed of the model while training^33^. Meanwhile, a regularization term is added to the objective function to control the complexity of the tree to obtain a simpler model and avoid overfitting^34^. In summary, XGBoost is a flexible and highly scalable tree structure enhancement model that can handle sparse data, greatly improve algorithm speed, and reduce computational memory in very large-scale data training.

### Optimization of model parameters

#### Tuning RF

The complexity of a model is determined by its parameters, which are the key elements of a model. Finding the best combination of parameters is critical to optimizing the model. For RF, only two parameters need to be tuned. The first tuning parameter, ntree, controls the number of trees to grow. The second, mtry, controls the number of predictor variables randomly sampled as candidates at each split. For each model, ntree is increased from 100 to 3000 in steps of 10. For mtry, besides the default (one third of the number of predictor variables), the half and 1.5 times of default also were used. For example, the Sentinel had 18 predictor variables; three values were considered for mtry: 3, 6, and 9.

RF was performed in the R software environment using the package *randomForest*, and the *randomForest* package has two indices to measure variable importance: the first measure is the percent increase in mean square error (*%IncMSE*), which is computed from permuting OOB data; and the second measure is the total decrease in node impurities from splitting on the variable (*IncNodePurity*), which is measured by residual sum of squares^35^.

#### Tuning XGBoost

The most important parameters of XGBoost include: (1) *nrounds*, which is the maximum number of boosting iterations. (2) *max_depth* is the maximum depth of an individual tree. (3) *min_child_weight* is the minimum sum of instance weight needed in a leaf node. (4) *gamma* is the minimum loss reduction required to make a further partition on a leaf node of the tree. (5) *subsample* is the subsample ratio of the training instances or rows. (6) *learning_rate* is used during updating to prevent overfitting.

Tuning the XGBoost model is complicated because changing any one of the parameters can affect the optimal values of the others. Therefore, most previous studies used the default value of parameters for modeling, and few studies have described the details of the process of tuning the XGBoost parameters. Climent *et al*. used the non-default value of parameters in their XGBoost model and obtained a high-performance model, but the tuning process was not described^36^. Carmona *et al*. described the influence of tuning parameters on the performance of models, and the results demonstrated that it is crucial to tune the parameters in XGBoost^37^.

The grid search approach was used to find the best combination of parameters. The range of parameters for searching for the best combination set is as follows: *max_depth* is from 2 to 10 with 2 steps, *min_child_weight* is from 1 to 5 with 1 step, *gamma* is from 0 to 0.4 with 0.1 step, *subsample* is from 0.6 to 1 with 0.1 step, and *learning_rate* values are 0.01, 0.05, 0.1, 0.2, and 0.3. XGBoost was implemented by the R package *xgboost*, and the *xgboost* package has two indices to evaluate the variable importance, first by computing the fractional contribution of each feature to the model based on the total gain of this variable’s splits (*Gain*), and second by computing the relative number of times a feature be used in trees (*Frequency*)^38^.

### Evaluation of models

In this study, 10-fold cross-validation approach, which was suggested by Tyralis *et al.*^31^, was performed to validate the performance for the LR, RF and XGBoost models. Three error measurements, namely, the coefficient of determination (R^2^), the root mean square error (RMSE), and the percentage root mean square error (RMSE%), were used to evaluate the performance of models^18^. In general, a higher R^2^ value and lower RMSE and RMSE% values indicate a better estimation performance of the model.

## Results

### Evaluation of LR

The Pearson correlation coefficients between the predictor variables and AGB were calculated, and 52 variables had a significance level of 0.01 with the AGB. This result indicated that the Landsat 8 texture image variables had significant correlation with the AGB. Also, the correlation coefficients of Landsat 8 parameters were significantly higher than Sentinel-1A parameters. The variables with the highest correlation coefficient were *LT_B4_MEA* and *LT_B4_VAR*, both with a value of −0.38.

The LR models of three datasets (i.e., the Landsat 8, Sentinel-1A, and the combined dataset) were developed using the predictor variables, which were selected by stepwise regression (Supplementary Tables S3, S4). Thirteen models were established. The results indicated that the performance of the models was improved when the number of predictor variables increased and the range of predicted AGB values was extended to approach the observed AGB values, although the mean predicted AGB values were the same (49.44 Mg/ha). Model No. 13 with the combined dataset had the best performance with a R^2^, RMSE and RMSE% values of 0.22, 29.98, and 60.64, respectively.

The three best LR models (i.e., Model Nos. 6, 7, and 13) for the different datasets were selected as the base and was used to compare the performance of other types of models (Supplementary Table S4). The VIFs of the predictor variables were less than 2, which showed that the selected variables were effective. The predictor variables of these models were dominated by the image texture information. The standardized coefficients and the significance levels of the models showed that the texture-type variables contributed more than other type variables, which indicated that the texture variables were very important for the AGB estimation using the LR model in this study.

### Tuning process of RF and XGBoost

#### Tuning the RF models

The results indicated there was no evidence that overfitting occurs with the increase in the number of the trees (Supplementary Fig. S1). As the number of the trees increases, the RMSE decreases and then tends to be stable. We also found that the influence of *mtry* is higher when the models have a small number of trees, but the influence decreased when the number of trees increased.

For Landsat 8, the *mtry* value of 10 had the highest R^2^ and the lowest RMSE. The value of R^2^ went from 0.60 to 0.61 and RMSE went from 22.40 to 22.50, and were relatively steady with increasing the number of trees. For Sentinel-1A, the relationship of three lines of R^2^ was complex, but the lines of RMSE were easy to identify, and the values with the *mtry* of 3 were relatively lower. The value of R^2^ and RMSE were stabilized at 0.27 to 0.28 and 29.40 to 29.50, respectively. The model for the combined dataset with the *mtry* value of 13 had the best performance, and the value of R^2^ and RMSE were stabilized at 0.67 to 0.68 and 20.90 to 21.00, respectively. Therefore, the accuracy of RF models using the combined data of Landsat 8 and Sentinel-1A synergy was clearly improved.

The results also indicated that the performance of the models was stable after approximately 2000 trees, which was a high value of the number of trees in this study (Supplementary Fig. S1). Therefore, we selected the value of *ntree* with 2900, which was the highest value in the stable-tuning intervals. The accuracy of RF models using the combined data of Landsat 8 and Sentinel-1A synergy was clearly improved. The parameters of the optimized models are shown in Supplementary Table S5.

#### Tuning the XGBoost models

The tuning results show that XGBoost models are relatively insensitive to the *gamma* value, and that the values of R^2^ and RMSE did not have a significant difference at the same value of minimum child weight and maximum depth (Supplementary Fig. S2). As the maximum depth value of a tree increases (models more complex and more likely to overfit), a higher value of child weight is required to balance the resulting reduction of accuracy.

A lower learning rate is generally preferable. With a lower value of learning rate, which decreases the contribution of each tree, the models are more conservative in preventing overfitting, and thus more iterations are needed to reach the same accuracy. Compared with the highest accuracy of the optimized model, the models with a higher learning rate (learning rate = 0.2, 0.3) will overfit before reaching the highest accuracy (RMSE initially decreases quickly as iterations increase, but rises again with larger iterations). The results showed that the learning rate value of 0.01 had the best performance for the Landsat 8 dataset (Supplementary Fig. S3).

With smaller values of the subsample rate, the models with same learning rate needed more iterations to reach the overfitting points, and the performance of the model is better. The result indicated that the subsample rate values were lower than the default value (1.0) for the optimized models in this study. The subsample rate values were 0.7, 0.9, and 0.8 of the Landsat 8, Sentinel-1A, and the combined dataset, respectively. The results also shown that the model gained a good performance with a small value of *nrounds*, and then was approximately stable with the increase in the number of boosting iterations (Supplementary Fig. S3). It is crucial to select a suitable value of *nrounds*, which is very different than the RF model.

The relationship between learning rate, subsample rate, and model accuracy is stable. Therefore, it is reasonable to optimize the combination of the learning rate and the subsample rate. The above parameter optimization method is also applicable to the Sentinel-1A and combination datasets. Supplementary Table S6 shows the parameters of the optimized models.

### Comparative analysis of AGB models

#### Performance of the optimized models

**Figure 3:**
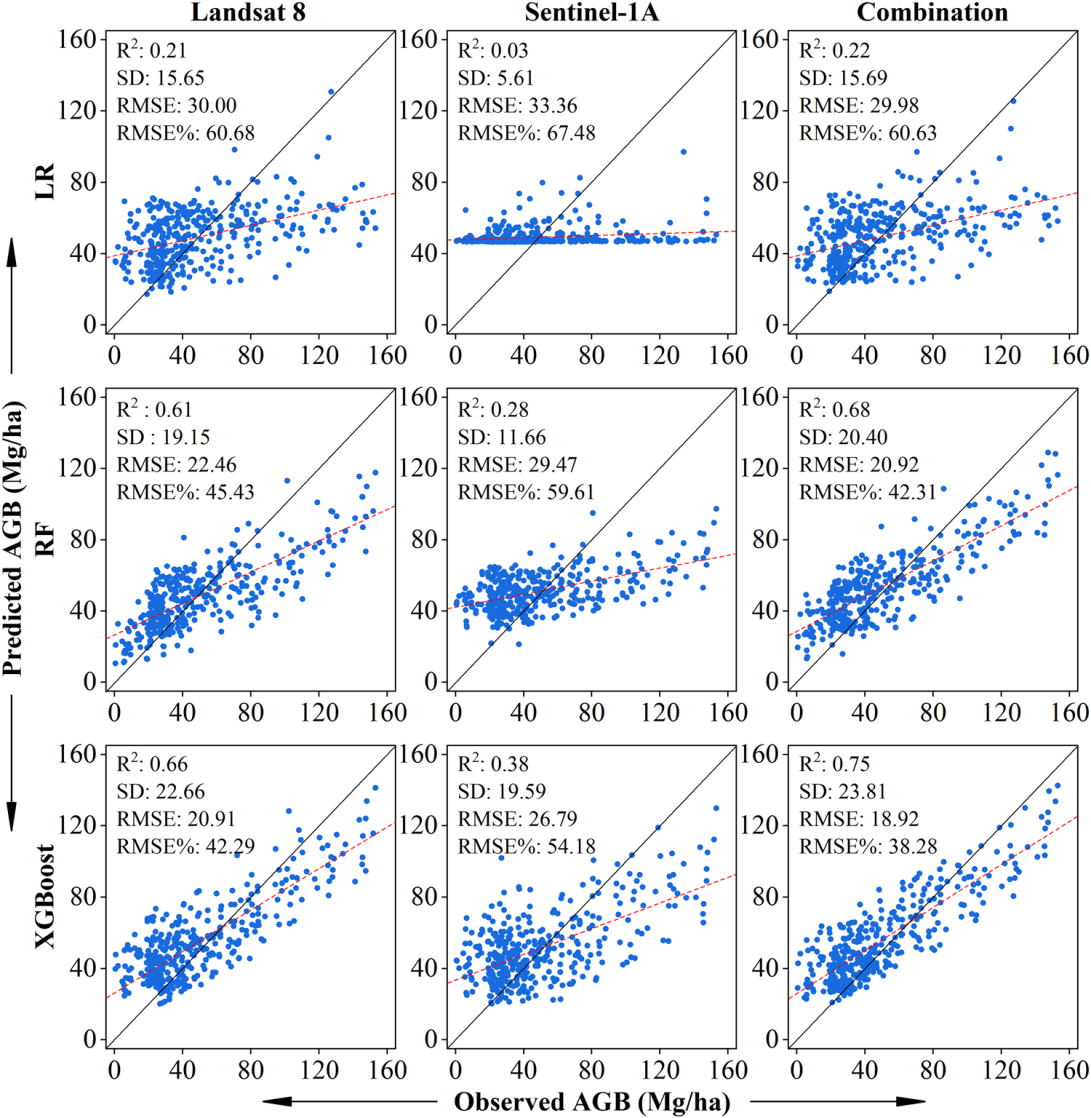
The predicted results of the LR, RF, and XGBoost models of the different datasets.

After the tuning parameters, we obtained the nine best models of the LR, RF, and XGBoost using the three different datasets. The performance of models could be explained by the scatterplots, which show the relationship between the observed AGB values and predicted AGB values (Fig. 3). They showed that the RF and XGBoost models worked better than the LR models with the same dataset. The SD values of the XGBoost and RF models were higher than LR, which means that the distributions of the predicted AGB values of the XGBoost and RF were more dispersed. And the XGBoost model worked slightly better than the RF model. Meanwhile, the results also indicated that the performance of models of the combined dataset, which synergize two datasets of Landsat 8 and Sentinel-1A, was not improved much more than the single dataset of Landsat 8. In contrast, the performance of the Sentinel-1A models was relatively poor.

We also found that the problems of overestimation and underestimation were experienced by all models. Intuitively, the predicted value is higher than the centerline when the biomass is low (AGB ≤ 20 Mg/ha) but lower when it is high (AGB ≥ 90 Mg/ha) in the figure. This means that the models still overestimate at the lower AGB values and underestimate at the higher AGB values although we used multi-sensor dataset synergy to reduce the estimation error.

To further verify whether the models had a significant difference between each other, the F-test was used (Supplementary Fig. S4). The confidence level was set at 95%. The F-test results showed that there were significant differences between the XGBoost and LR models; the RF and LR models were significantly different, except for the RF models using the Landsat 8 dataset. Between the XGBoost and RF models, there was also a significant difference except for the XGBoost models of the Sentinel-1A and combined datasets. It was note that the XGBoost models with the combination and Landsat 8 datasets had the highest accuracy of predicted AGB, but there was not a significant difference between them. It means that the contribution of Sentinel-1A dataset was weak for the model and the multi-sensor dataset synergy cannot significantly improve the performance of the XGBoost model.

#### Importance of predictor variables

Supplementary Fig. S5 shows the 15 most important predictor variables for the final optimized models of RF and XGBoost (see Supplementary Table S4 for the LR models). We expected that the important variables of the LR, RF, and XGBoost models would be similar, but our findings were different. In three datasets, the largest quantity of important variables were the texture variables, indicating that the texture images, especially Landsat texture images, have abundant information to promote the performance of models for biomass estimation^6^. For Landsat 8, the most important variables of the LR, RF, and XGBoost models were *LT_B4_MEA*, *LT_B2_MEA*, and *LT_B4_MEA*, respectively, and the texture variables were dominant; but it should be noted that the spectral variables were also very important in the XGBoost. For Sentinel-1A, the cross-polarization variables are more important to the models^10^. For the combined dataset, the Landsat variables are more important than the Sentinel-1A variables, and the model results showed that the performance of models was not significantly improved, although the Sentinel-1A variables were added. We found that RF models split the importance to the correlated multiple variables, whereas XGBoost models are inclined to centralize the importance at a single variable (Supplementary Tables S7, S8). For example, *LT_B4_MEA* is significantly correlated with *LT_B2_MEA* at a significance level of 0.01; for the Landsat dataset, they had a similar importance in the RF model, but the importance is concentrated on *LT_B4_MEA* in the XGBoost model. This conclusion is the same as that reported by Freeman *et al*.^23^.

### Mapping AGB

We estimated the AGB of the study area using the LR, RF, and XGBoost models of the combined dataset with the optimal parameters, then calculated the difference between them. We also created the histograms of predicted AGB and their difference.

**Figure 4:**
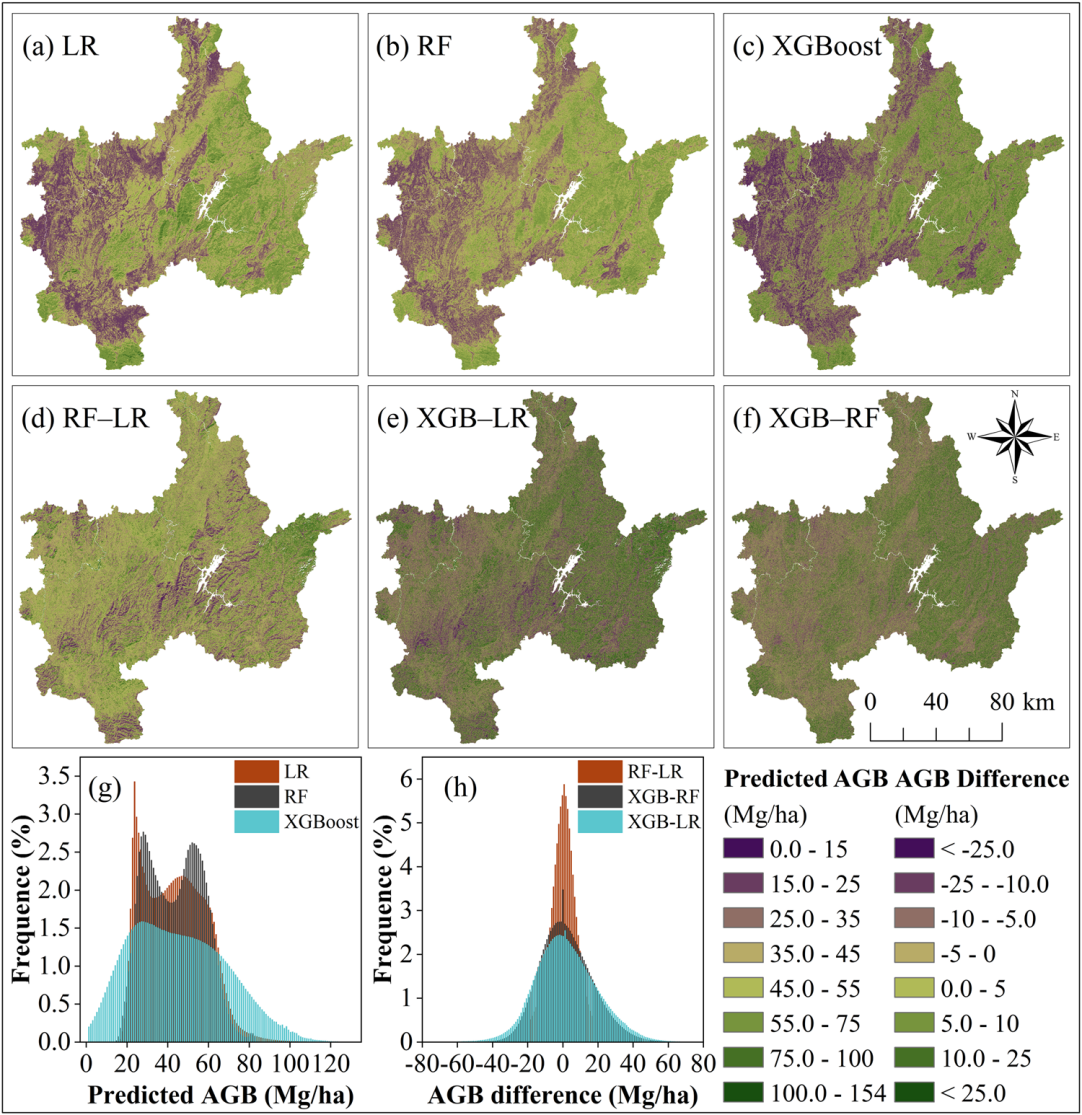
The predicted AGB using (**a**) LR, (**b**) RF and (**c**) XGBoost models with the optimal parameters, including (**d**) the difference between RF and LR (RF ‒ LR), (**e**) the difference between XGBoost and LR (XGBoost ‒ LR), (**f**) the difference between XGBoost and RF (XGBoost ‒ RF), (**g**) the histograms of pixel values frequency of LR, RF and XGBoost models, and (**h**) their difference.

In the study area, the LR, RF, and XGBoost predictions had a similar trend in spatial distribution. As shown in Fig. 4, the AGB value is high in the east and low in west. This is consistent with the AGB distribution trend of the inventory plots in Fig. 1 because of the eastern area has a high altitude, high vegetation coverage, and less human interference, while the conditions in the western area are the opposite. However, the ranges of predicted AGB by the three models were different. The values were from 0.52 to 153.96 Mg/ha by the XGBoost model, and the distribution of its histogram was similar to the inventory values in Fig. 1. However, the ranges of values were from 11.32 to 93.84 Mg/ha and from 16.76 to 143.72 Mg/ha by the LR and RF models, respectively. The histogram of XGBoost predictions was a unimodal distribution, which approximated a normal distribution (Fig. 4(g)). Compared with XGBoost, the main intervals of the distribution of the LR and RF were similar, and they were all a bimodal distribution, but the details were different. The frequencies of the LR were very high at the range of predicted AGB from 22 to 27 Mg/ha; in this range, the points were also very dense in the scatterplot in Fig. 4, but the histogram of RF predictions was more symmetrical than LR. The histogram also indicted that the ability of the LR and RF estimations were clearly insufficient at the high and low values. In Fig. 4(h), the histogram of the AGB difference between the RF and LR was very concentrated, and other histograms were dispersed as a normal distribution, which also indicated that the XGBoost model was clearly different than the RF and LR models.

**Figure 5:**
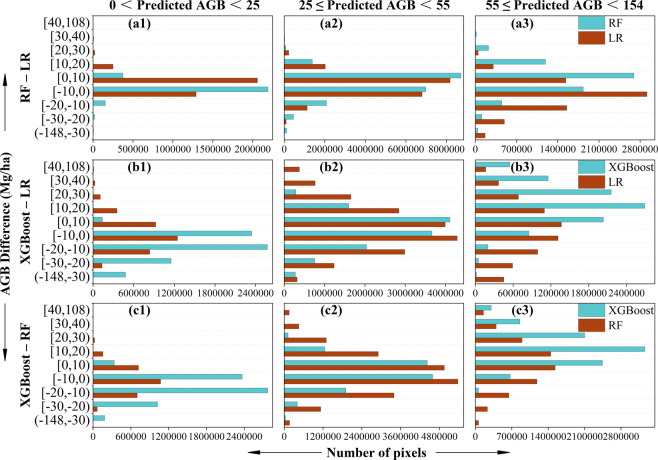
Histograms illustrating the difference of pixel numbers in three ranges. Note that the vertical axis labels represent the range of the prediction difference between XGBoost, RF, and LR.

The degrees of overestimation and underestimation of the three models were different. To further verify this conclusion, we sorted the values of the predicted AGB into three ranges: low (0 ≤ predicted AGB < 25), medium (25 ≤ predicted AGB < 55), and high (55 ≤ predicted AGB ≤ 154) values (Fig. 5). The corresponding values of predicted AGB and AGB difference were obtained by the overlay operations. In the low range of predicted AGB, most of the values of XGBoost predictions were less than the LR and RF predictions (Fig. 5(b1,c1)), but the values of the RF and LR difference were distributed from -10 to 10 Mg/ha (Fig. 5(a1)), which did not have an obvious difference; therefore, the XGBoost model had the best predicted performance. In the medium range, the distribution of the AGB difference approximated a normal distribution, indicating that the three models have similar performance for medium values of AGB (Fig. 5(a2,b2,c2)). In the high range, the XGBoost predicted values were clearly larger than those of LR and RF, indicating that the XGBoost model had the best performance at the high AGB values (Fig. 5(a3,b3,c3)). In summary, the XGBoost model can better estimate AGB value than the LR and RF models whether in the high or low AGB.

## Discussion

### Importance of the tuning process

Through the tuning process, we increase our understanding of the selection of parameters for the RF and XGBoost models. Tuning is sometimes overlooked in modeling processes. The default value of parameters was also used while ignoring the difference of datasets between different study areas. For nonparametric models, it is especially important to optimize the model parameters. Our research results show that parameter optimization is particularly important for XGBoost models.

XGBoost models have a large number of parameters, and the performance of models can vary greatly depending on the selected values of these parameters. XGBoost models are also more susceptible to overfitting if proper parameter values are not chosen. For XGBoost models, deeper trees generate a more complex model, capture too many details, and overfit the training dataset, leading to the decline of the generalization ability of the model. The model cannot make good predictions on a new dataset. We found that larger child weight was needed to make the models reach a point of increasing returns (Supplementary Fig. S2). Thus, choosing the appropriate combined values of tree depth and child weight was an important step to improve the quality of the models. The minimum loss reduction, *gamma*, controls the splitting complexity of a tree leaf node, which is split only when the resulting split give a positive reduction in the loss function, and a larger value of *gamma* makes the models more robust to avoid overfitting^32^. *gamma* is a very important parameter of XGBoost model, although its effect was not significant in this study (Supplementary Fig. S2).

Generally, a lower learning rate usually improves the performance of models, but there is a point of diminishing returns, and the model performance gradually stabilizes after this point. To a certain degree, learning rate is negatively corelated to the number of iterations, so the models need more iterations to reach the same accuracy with a lower value of learning rate (Supplementary Fig. S3). With a higher learning rate, the performance of models can reach the optimum point quickly and then decrease quickly with the increasing number of iterations. Therefore, increasing iterations beyond a proper range with a higher learning rate selection does not improve the quality of the model and increases the calculation cost. A lower learning rate reduces the influence of each individual tree and leaves space for feature trees to improve the performance of the model.

For the subsample rate, Freeman *et al*.^23^ found the best model had a subsample rate near 0.5, and Elith *et al*.^39^ suggested selecting a value between 0.5 and 0.75. In contrast, the subsample rate values were higher than the suggested values for the optimized models in this study (Supplementary Table S6). A smaller subsample ratio, which can also collect enough samples from a large training dataset, can add more stochasticity into the model to offset overfitting. However, for a small dataset, the model performance will decline with a very low subsample ratio that cannot provide enough information to build trees. In addition, the XGBoost model supports the column subsampling (i.e., variable subsampling), which is also used in the RF model. According to user feedback, using the column subsampling prevents overfitting even more than the row subsampling, and also speeds up computations of the parallel algorithm described later^32^.

The number of boosting iterations (*nrounds*) is particularly important in XGBoost. The boosting tree of a model is built sequentially, where each new tree attempts to correct the error made by the previous trees. The model can quickly reach a point of diminishing returns. Therefore, the performance of the model will not improve when more trees are added beyond a limit (Supplementary Fig. S3).

XGBoost has a lot of parameters besides the parameters introduced in this study, and the relationship between these parameters is complicated. Undoubtedly, it is difficult to totally understand the interaction between these parameters. However, we are all delighted at the significant improvement of the estimation using the XGBoost, and this is also what we desired, although it takes a lot of time to tune the parameters. We also noticed that in XGBoost, many parameters need to be tuned. For researchers who are not familiar with how XGBoost works and meaning of the parameters, and it will take a lot of time to find the optimal values of parameters, which may affect the application of XGBoost in forest biomass estimation based on remote sensing data. Because of the large number of parameters and flexibility of XGBoost, there is not a fixed step of parameter optimization for different datasets. Therefore, we introduced a general approach for parameter tuning: first, choose a relatively high learning rate (*learning_rate*). Generally, a learning rate of 0.1 works, but somewhere between 0.05 to 0.3 should work for different problems. Then, determine the optimum number of trees (*nrounds*) for this learning rate. Second, tune tree-specific parameters, such as *max_depth*, *min_child_weight*, *gamma*, and *subsample*, for the decided learning rate and number of trees. Third, tune the regularization parameters for XGBoost, which can help improve running speed, reduce model complexity, and enhance performance. Fourth, lower the learning rate and decide upon the optimal parameters. Additionally, we should balance the improvement of model performance and the increased time consumption of parameter optimization according to the actual demand. The default values of XGBoost parameters are obtained by the developers through repeated tests. Compared with using the optimal parameters, although the model with the default values of parameters cannot get a best performance, it can greatly save the running time of the model, especially for large dataset. With the advantage of excellent performance of the XGBoost, by using the default values of parameters, we can quickly determine the dataset quality, the performance of the model, and determine whether to further optimize the parameters.

In contrast, the RF model has only two parameters that need to be optimized. Regarding the number of trees, Probst *et al*. proposed setting this parameter as high as possible^24^, and Liaw *et al*. suggested that a stabilized accuracy of the model after a specific number of trees can reliably express the performance of the model^40^. In practice, it is controversial “whether the number of trees should simply to be set to the largest computationally manageable value or whether a smaller value may in some cases be better, in which case the number of trees should ideally be tuned carefully”^24^. Our research results showed that the model prediction results were stabilized after a larger number of trees, which can realistically express the performance of the model. In addition, the trees of the RF model are independent of each other, which was different from the XGBoost model; thus, the RF model is less susceptible to overfitting when the number of trees increases^41^. The accuracy of the model was influenced by the value of *mtry*, and thus it needed to be carefully tuned. The results of the three values of *mrty* were clearly identified. However, we selected only three suggested values to tune, and other values also need to be tested. RF is a very important and remarkable non-parameter method although its performance was not superior to XGBoost in this study.

### Effectiveness of machine learning methods

Most early biomass estimation studies were based on classical statistical regression approaches, e.g., linear regression, which assumes a linear relationship between predictor and predicted variables^10,42^. However, there is a complex relationship between forest AGB and remote sensing data, so the traditional statistical regression method cannot fully describe the relationship between them. The machine learning methods such as random forest and gradient boosting can establish a complex non-linear relationship between vegetation information and remote sensing images with an indeterminate distribution of data, and can flexibly combine different sources data to improve the accuracy of the prediction^43,44^.

In this study, we have 78 predictor variables from different remote sensing images (Supplementary Table S2). We found that the synergy between machine learning algorithms and multi-sensor data can prevent overfitting and significantly improve the estimation accuracy compared with the traditional LR and RF models. The result indicated that the XGBoost model worked better than RF model (Fig. 3). The reason that XGBoost worked better is that besides XGBoost being a higher flexible algorithm and having the ability to correct the residual error to generate a new tree based on the previous tree, the trees are independent in the RF model^45^. The XGBoost model is an advanced GB system; it provides an improvements in processing a regularized learning objective, which can avoid overfitting^32^. It is noted that the problems of overestimation and underestimation were still not completely eliminated using machine learning algorithms. This problem also existed in the previous studies with a nonparametric algorithm for AGB estimation^46^. This is decided by the algorithm itself. The decision trees, which are the key components of the RF and XGBoost methods, cannot extrapolate outside the training set. In addition, there are the saturation problems of the biomass estimation when using the remote sensing dataset^47^. And the number of plots is not large enough, we have not made a stratified estimation based on biomass levels or forest types, which may reduce more estimation error.

Moreover, the issue of variable importance also needs to be concerned. It is well acknowledged that the importance of predictor variables can vary in different environments. Most of the study areas are mountainous and hilly, with a wide range of altitude changes. As the altitude changes, the temperature and precipitation also change, resulting in a variety of vegetation forms in the study area. Therefore, the texture features were abundant and easy to be identified in the remote sensing images. Despite the importance of the texture predictor variables were different from the previous studies, there is an undeniable importance of the texture images (Supplementary Fig. S5)^3,18^. In addition, based on the variable importance, variables can be selected from the high-dimensional dataset, which has a complex relationship between variables^48^. Variable selection, which can alleviate the effects of noise and redundant variables, is an important step in machine learning algorithms, but we did not perform this process. The reason is that our dataset does not have too many variables, and more importantly, our goal is to compare the performance of the models between a single sensor dataset and multi-sensors combined dataset, but all Sentinel variables may be lost after multiple rounds of variable selections. In addition, the parallel ensemble is usually noise-resistant; thus, the RF algorithm is not influenced by including the model noisy predictor variables^49^; the XGBoost model could restrain the noise predictor variables by the regularization objective, although it is more sensitive to noise than RF^50^.

Before this study, few studies had used the XGBoost algorithm to estimate AGB and described the process of parameter optimization. Gao *et al*.^51^ used multiple machine learning algorithms (KNN, ANN, SVM, RF) to estimate AGB for the subtropical forest, and the result indicated the machine learning algorithms had a good performance for AGB estimation. Li *et al*.^18^ used a linear dummy variable model and linear mixed-effects model to estimate AGB in the western Hunan Province of China; the R^2^ of total vegetation both were 0.41, and the R^2^ of the LR model using the combined dataset was only 0.22. In contrast, the results obtained by machine learning methods in this study were better, and the XGBoost algorithm had a good performance in AGB estimation and could reduce underestimation and overestimation to some extent.

In this study, we found that the average AGB of the study area is lower than that of the typical subtropical forest (Fig. 1). In the study area, there is a large proportion of secondary forests, and the distribution of age classes is uneven; it was composed of young and middle age forests, with few dominant and mature and over-mature forests. For forests with a high AGB value, saturation is the main reason for AGB underestimation based on remote sensing data. Because of the small number of plots with a high AGB value in this study, this problem is not fully reflected. How to improve the estimation accuracy of high AGB forest is still an important topic to be addressed in the future research. Li *et al*. established a linear dummy variable model to improve the AGB estimation accuracy according to the difference of forest canopy density^18^; Li *et al*. built an estimation model based on forest type to improve the AGB estimation accuracy^25^. In addition, remote sensing data with higher spatial and radiometric resolution, such as LIDAR data and hyperspectral data, or the approach of mixed pixel decomposition, data cleaning, and variable selection, may be solutions for AGB estimation.

### Potential of optical and SAR images synergy for AGB estimation

Optical images have been applied to estimate forest AGB in the earlier studies, but the previous results showed that the penetrability of optical signals is weak. Therefore, the optical signals mainly record the horizontal structure of the forest^52^. However, the forest AGB is dominated by stem and branch biomass, so the optical data obviously shows its drawbacks for forest AGB estimation. For a specific wavelength of microwave, the SAR signals can penetrate forest canopy and record the vertical structure information, especially the long wave SAR, such as L-band and P-band, has the stronger penetration ability. However, the SAR signal is susceptible to terrain and is easily saturated when the canopy density is too high or biomass is too large, which decreases the accuracy of forest structure parameters extraction^10,11^. Therefore, using the combination of optical and SAR data to extract parameters for forest AGB estimation is a new approach.

The recent research proved that it is an effective way to improve the accuracy in multi-sensor data synergy for AGB estimation. Shen *et al*. mapped the subtropical forest AGB data by combining the Landsat TM/ETM + and ALOS L-band SAR images in Guangdong Province of China, and the result shown that the multi-sensor image-based AGB correlated well^53^. Cutler *et al*. used Landsat TM and JERS-1 L-band SAR together to estimate tropical forest biomass at three separate geographical locations, and the result indicated the data fusion could improve the accuracy of biomass estimation^54^.

In this study, the results demonstrated that the combined dataset works better than single remote sensing data, despite the Sentinel-1A data does not work as a good predictor variable for the study area due to its relatively low penetration ability. Meanwhile, it also proved that the feasibility of Landsat 8 and Sentinel-1A, which are two important free remote sensing systems used for global monitoring and observation, can be combined together for forest AGB estimation. They both are a component (the latest or the earliest) of a series missions, thus the continuous data can help to establish a consistent and long-term archive for continuous observation, which is why we chose these two remote sensing datasets in this study.

## Conclusions

This study selected the subtropical region of southeastern Hunan Province, China, as a case study area to analyze the AGB estimation based on Sentinel-1, Landsat 8, and their combination using different modeling algorithms, LR, RF and XGBoost. The results indicate the following: (1) Parameter optimization is a very important part of the machine learning methods. In this study, parameter tuning had a significant effect on the performance of XGBoost over that of RF. After tuning, the R^2^ of the XGBoost model reached 0.75. (2) Machine learning algorithms have advantages in biomass estimation. Although there are still problems of high value underestimation and low value overestimation for these two algorithms, the XGBoost algorithm reduced this problem to a certain extent and made the AGB estimation results closer to the sample survey results. Meanwhile, the RF and XGBoost models significantly improved compared with the LR model. (3) Compared with Sentinel-1A, the Landsat 8 image had a more accurate estimation of AGB, and their combination was helpful for improving the AGB estimation. Therefore, the combination of the two datasets can be effectively used in regional biomass estimation. To date, Landsat 8 and Sentinel-1 data are available at no cost and provide high spatial, spectral, and temporal resolution data. In addition, we have described in detail the modeling process of the two algorithms in this study. The software and data involved in the article are free or can be applied for trial, which is convenient for other researchers, especially young students, to repeat easily and not worry about the cost. Hopefully, these results will encourage more researchers to take full advantage of these data and explore more efficient models for biomass estimation. In the future, we will explore using other sources of data, such as longer band SAR, LiDAR, hyperspectral, high spatial resolution remote sensing images, and auxiliary data (e.g., climate, soil, and DEM), for AGB estimation.

## Supplementary information

Supplementary information.

## Data Availability

The numerical datasets generated and analyzed during the current study are available from the corresponding author on reasonable request.
