# Supplementary material for: Forest aboveground biomass estimation using Landsat 8 and Sentinel-1A data with machine learning algorithms
Source: Sci Rep. 2020 Jun 19;10:9952. doi: 10.1038/s41598-020-67024-3 (PMC7305324; doi:10.1038/s41598-020-67024-3)
Supplement: Supplementary file 1 — Supplementary information. [file 41598_2020_67024_MOESM1_ESM.docx]

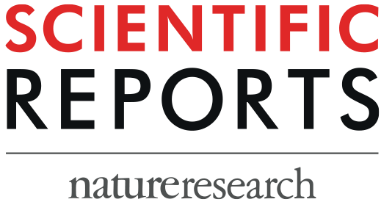


Forest aboveground biomass estimation using Landsat 8 and Sentinel-1A data with machine learning algorithms

**Yingchang Li^[[1]](#footnote-1)^, Mingyang Li^1^, Chao Li^1^ and Zhenzhen Liu^2^**

**Supplementary Information**





**Figure S1.** Tuning RF and the influence of *mtry* and *ntree* values on RF models for three datasets. Each line represents the change of one model as the number of the trees is increased, and each color indicates a different *mtry* value.





**Figure S2.** Tuning XGBoost and the influence of maximum depth of a tree (*max_depth*), minimum sum of instance child weight (*min_child_weight*), and minimum loss reduction (gamma) on XGBoost models of Landsat 8 dataset. Models are relatively insensitive to gamma value, and *max_depth* and *min_child_weight* have a stable relationship.





**Figure S3.** Tuning XGBoost and the effect of learning rate and subsample ratio of the training instances on XGBoost models using the Landsat 8 dataset.


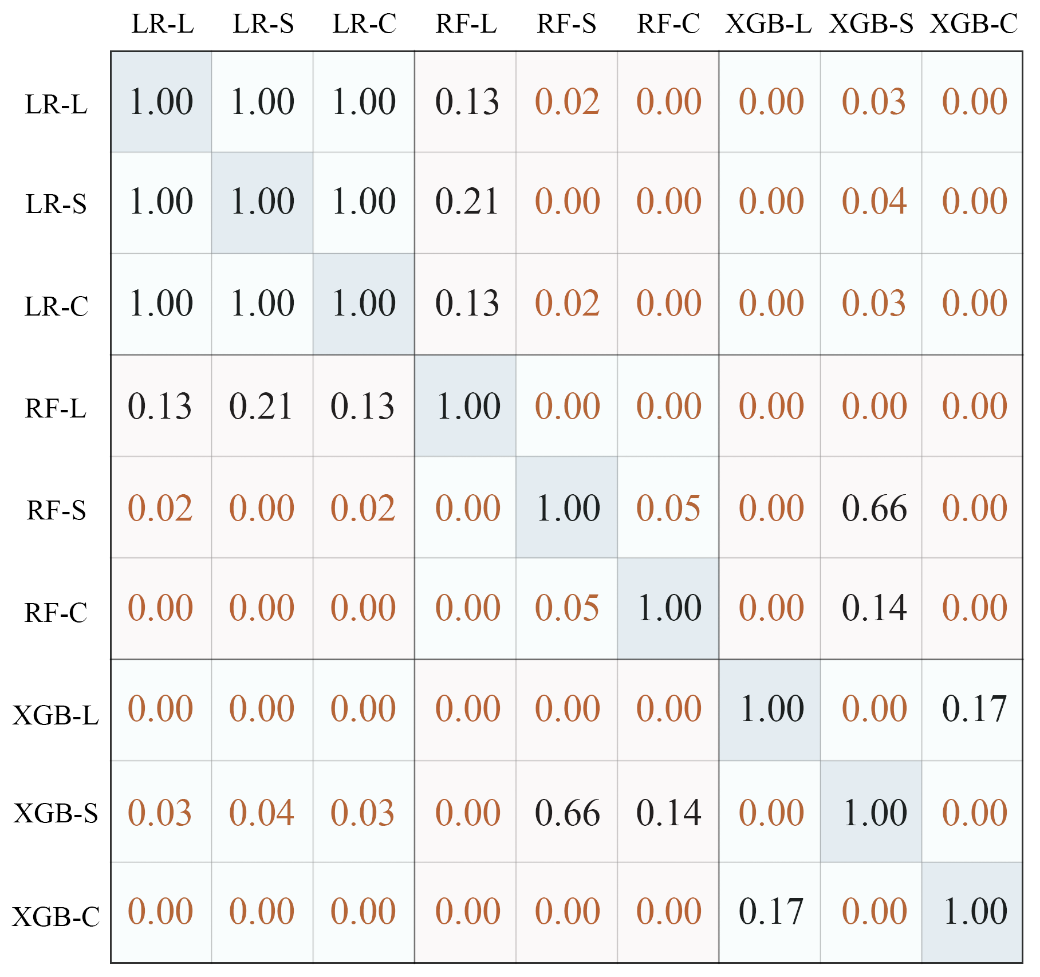


**Figure S4.** The comparisons of the LR, RF, and XGBoost models. The numbers are the p-values, which are from the F-test. The orange numbers indicate a significance level of 0.05. The labels of the vertical and horizontal axes represent the models using different dataset; L, S, and C represent the Landsat 8, Sentinel-1A and the combination datasets, respectively; and XGB represents the XGBoost model.





**Figure S5.** The most important predictor variables for the final optimized models of RF and XGBoost. A higher value of these measures means a more important predictor variable.

**Table S1.** Remote sensing data used in this study.

| **Remote Sensing Data** | **Path, Frame/ Row** | **Acquisition Date** | **Scene Cloud Cover (%)** | **Usage** |
| --- | --- | --- | --- | --- |
| Sentinel-1A | P11, F74 | October 13, 2015 | – | Backscatter and texture variables |
|  | P11, F79 | October 13, 2015 | – |  |
|  | P113, F80 | October 20, 2015 | – |  |
|  | P113, F85 | October 20, 2015 | – |  |
| Landsat 8 Operational Land Imager | P122, R42 | October 18, 2015 | 0.02 | Spectral and texture variables |
|  | P122, R43 | October 18, 2015 | 0.03 |  |
|  | P123, R41 | October 25, 2015 | 0.96 |  |
|  | P123, R42 | October 25, 2015 | 18.36 |  |
|  | P123, R43 | October 25, 2015 | 22.83 |  |

**Table S2.** Summary of predictor variables including Landsat 8 spectrum, vegetation index, Sentinel backscatter, and texture of Landsat 8 and Sentinel-1A for AGB estimation models.

| **Variable Type** | **Variable Name** | **Number of Variables** | **Description** |
| --- | --- | --- | --- |
| Sentinel backscatter | SB_VV, SB_VH | 2 | Sentinel-1A dual polarization backscatter |
| Sentinel texture | ST_PP_CON, ST_PP_DIS, ST_PP_MEA, ST_PP_HOM, ST_PP_ASM, ST_PP_ENT, ST_PP_VAR, ST_PP_COR, … | 16 | Sentinel-1A backscatter (VV and VH) texture measurement using gray-level co-occurrence matrix |
| Landsat band reflectance | LR_B2, LR_B3, LR_B4, LR_B5, LR_B6, LR_B7 | 6 | Landsat 8 Bands 2–7 |
| Landsat vegetation index | LV_NDVI  LV_SAVI  LV_DVI  LV_RVI  LV_ARVI  LV_EVI | 6 | Normalized difference vegetation index (NDVI)  Soil adjusted vegetation index (SAVI)  Difference vegetation index (DVI)  Ratio vegetation index (RVI)  Atmospherically resistant vegetation index (ARVI)  Enhanced vegetation index (EVI) |
| Landsat texture | LT_Bi_CON, LT_Bi_DIS, LT_Bi_MEA, LT_Bi_HOM, LT_Bi_ASM, LT_Bi_ENT, LT_Bi_VAR, LT_Bi_COR, … | 48 | Landsat bands 2–7 texture measurement using gray-level co-occurrence matrix |

Note: *LT_Bi_XXX* represents a texture image developed on the Landsat band *i* (2–7) using the texture measure *XXX*, where *XXX* is CON (contrast), DIS (dissimilarity), MEA (mean), HOM (homogeneity), ASM (angular second moment), ENT (entropy), VAR (variance), or COR (correlation). *ST_PP_XXX* represents a texture image developed on the Sentinel-1A image using the texture measure *XXX*, where *PP* is either VV or VH.

**Table S3.** Error measurements of the LR models using Landsat 8, Sentinel-1A, and the combined dataset.

| **Dataset** | **Model No.** | **Number of predictor variables** | **R^2^** | **RMSE** | **RMSE%** | **Standard error** | **Predicted maximum** | **Predicted minimum** |
| --- | --- | --- | --- | --- | --- | --- | --- | --- |
| Landsat 8 | 1 | 1 | 0.15 | 31.27 | 63.25 | 31.35 | 79.09 | 31.35 |
|  | 2 | 2 | 0.16 | 30.95 | 62.60 | 31.08 | 103.96 | 26.90 |
|  | 3 | 3 | 0.17 | 30.77 | 62.24 | 30.94 | 99.13 | 25.39 |
|  | 4 | 4 | 0.19 | 30.49 | 61.67 | 30.70 | 119.20 | 25.65 |
|  | 5 | 5 | 0.20 | 30.20 | 61.08 | 30.45 | 124.01 | 18.89 |
|  | 6 | 6 | 0.21 | 30.00 | 60.68 | 30.29 | 130.69 | 17.27 |
| Sentinel-1A | 7 | 1 | 0.03 | 33.36 | 67.48 | 33.45 | 96.95 | 46.56 |
| Combination | 8 | 1 | 0.15 | 31.27 | 63.25 | 31.35 | 79.09 | 31.35 |
|  | 9 | 2 | 0.16 | 30.95 | 62.60 | 31.08 | 103.96 | 26.90 |
|  | 10 | 3 | 0.17 | 30.74 | 62.18 | 30.91 | 104.50 | 24.99 |
|  | 11 | 4 | 0.18 | 30.57 | 61.83 | 30.78 | 99.86 | 23.67 |
|  | 12 | 5 | 0.20 | 30.25 | 61.19 | 30.50 | 120.97 | 23.77 |
|  | 13 | 6 | 0.22 | 29.98 | 60.64 | 30.27 | 125.55 | 18.87 |

**Table S4.** The predictor variable estimation of the best selected LR model using Landsat 8 (Model No. 6), Sentinel-1A (Model No. 7), and the combined dataset (Model No. 13).

| **Dataset** | **Predictor variable** | **Standardized coefficients** | **Estimate (t-test)** | **Significance (p-value)** | **Collinearity statistics** |
| --- | --- | --- | --- | --- | --- |
| Landsat 8 | LT_B4_MEA | -0.46 | -7.99 | 0.00 | 1.51 |
|  | LT_B2_CON | 0.21 | 3.93 | 0.00 | 1.25 |
|  | LT_B2_COR | 0.22 | 3.59 | 0.00 | 1.68 |
|  | LT_B5_COR | -0.20 | -3.62 | 0.00 | 1.43 |
|  | LV_RVI | 0.13 | 2.53 | 0.01 | 1.18 |
|  | LR_B5 | 0.11 | 2.19 | 0.03 | 1.18 |
| Sentinel-1A | ST_VH_CON | 0.17 | 3.21 | 0.00 | 1.00 |
| Combination | LT_B4_MEA | -0.43 | -7.63 | 0.00 | 1.46 |
|  | LT_B2_CON | 0.21 | 3.97 | 0.00 | 1.25 |
|  | SB_VV | 0.11 | 2.31 | 0.02 | 1.01 |
|  | LT_B2_COR | 0.21 | 3.44 | 0.00 | 1.68 |
|  | LT_B5_COR | -0.18 | -3.30 | 0.00 | 1.33 |
|  | LV_RVI | 0.13 | 2.59 | 0.01 | 1.17 |

**Table S5.** The parameters of the optimized models and error measurements using Landsat 8, Sentinel-1A, and the combined dataset on the RF model.

| **Type** | **Name** | **Landsat 8** | **Sentinel-1A** | **Combination** |
| --- | --- | --- | --- | --- |
| Parameter | ntree | 2900 | 2900 | 2900 |
|  | mtry | 10 | 3 | 13 |
| Error measurement | R^2^ | 0.61 | 0.28 | 0.68 |
|  | RMSE | 22.46 | 29.47 | 20.92 |
|  | RMSE% | 45.43 | 59.61 | 42.31 |

**Table S6.** The parameters of optimized models and testing error measurements for Landsat 8, Sentinel-1A, and the combined dataset on the XGBoost model.

| **Type** | **Name** | **Landsat 8** | **Sentinel-1A** | **Combination** |
| --- | --- | --- | --- | --- |
| Parameter | max_depth | 6 | 10 | 10 |
|  | min_child_weight | 2 | 5 | 3 |
|  | gamma | 0.4 | 0.2 | 0.2 |
|  | subsample | 0.7 | 0.9 | 0.8 |
|  | learning_rate | 0.01 | 0.01 | 0.05 |
|  | nround | 410 | 320 | 375 |
| Error measurement | R^2^ | 0.66 | 0.38 | 0.75 |
|  | RMSE | 20.91 | 26.79 | 18.92 |
|  | RMSE% | 42.29 | 54.18 | 38.28 |

| **Table S7.** The correlation between the Landsat 8 variables, which were the most important predictor variables for the final optimized models of the LR, RF and XGBoost. | **LV_ARVI** |  |  |  |  |  |  |  |  |  |  |  |  |  | 1 | 0.29** | Note: ** and * indicate a significance level of 0.01 and 0.05, respectively. |
| --- | --- | --- | --- | --- | --- | --- | --- | --- | --- | --- | --- | --- | --- | --- | --- | --- | --- |
|  | **LT_B5_COR** |  |  |  |  |  |  |  |  |  |  |  |  | 1 | 0.09 | 0.06 |  |
|  | **LT_B4_MEA** |  |  |  |  |  |  |  |  |  |  |  | 1 | 0.28** | -0.70** | -0.27** |  |
|  | **LT_B4_HOM** |  |  |  |  |  |  |  |  |  |  | 1 | 0.48** | 0.03 | -0.61** | -0.07 |  |
|  | **LT_B3_MEA** |  |  |  |  |  |  |  |  |  | 1 | 0.49** | 0.97** | 0.28** | -0.60** | -0.27** |  |
|  | **LT_B3_HOM** |  |  |  |  |  |  |  |  | 1 | 0.41** | 0.88** | 0.40** | 0.02 | -0.51** | -0.08 |  |
|  | **LT_B3_COR** |  |  |  |  |  |  |  | 1 | 0.29** | 0.58** | 0.32** | 0.52** | 0.54** | -0.18** | -0.12* |  |
|  | **LT_B3_ASM** |  |  |  |  |  |  | 1 | 0.2** | 0.88** | 0.41** | 0.74** | 0.40** | 0.05 | -0.49** | -0.09 |  |
|  | **LT_B2_VAR** |  |  |  |  |  | 1 | 0.61** | 0.47** | 0.64** | 0.89** | 0.70** | 0.87** | 0.14** | -0.70** | -0.25** |  |
|  | **LT_B2_MEA** |  |  |  |  | 1 | 0.97** | 0.50** | 0.56** | 0.53** | 0.95** | 0.59** | 0.91** | 0.19** | -0.65** | -0.28** |  |
|  | **LT_B2_COR** |  |  |  | 1 | 0.49** | 0.37** | 0.14** | 0.83** | 0.15** | 0.49** | 0.16** | 0.41** | 0.41** | -0.13* | -0.25** |  |
|  | **LR_B6** |  |  | 1 | 0.21** | 0.51** | 0.50** | 0.30** | 0.32** | 0.26** | 0.65** | 0.33** | 0.69** | 0.29** | -0.45** | -0.15** |  |
|  | **LR_B4** |  | 1 | 0.81** | 0.28** | 0.76** | 0.77** | 0.49** | 0.38** | 0.49** | 0.78** | 0.58** | 0.81** | 0.20** | -0.73** | -0.23** |  |
|  | **LR_B2** | 1 | 0.91** | 0.62** | 0.46** | 0.88** | 0.85** | 0.46** | 0.51** | 0.47** | 0.84** | 0.54** | 0.81** | 0.19** | -0.64** | -0.27** |  |
|  | **AGB** | -0.29** | -0.29** | -0.20** | -0.12* | -0.35** | -0.34** | -0.17** | -0.27** | -0.16** | -0.36** | -0.18** | -0.38** | -0.18** | 0.27** | 0.18** |  |
|  | **Variable Name** | LR_B2 | LR_B4 | LR_B6 | LT_B2_COR | LT_B2_MEA | LT_B2_VAR | LT_B3_ASM | LT_B3_COR | LT_B3_HOM | LT_B3_MEA | LT_B4_HOM | LT_B4_MEA | LT_B5_COR | LV_ARVI | LV_RVI |  |

| **Table S8.** The correlation between the Sentinel-1A variables, which were the most important predictor variables for the final optimized models of the LR, RF and XGBoost. | **ST_VV_MEA** |  |  |  |  |  |  |  |  |  |  |  |  |  | 1 | 0.98** | Note: ** and * indicate a significance level of 0.01 and 0.05, respectively. |
| --- | --- | --- | --- | --- | --- | --- | --- | --- | --- | --- | --- | --- | --- | --- | --- | --- | --- |
|  | **ST_VV_HOM** |  |  |  |  |  |  |  |  |  |  |  |  | 1 | 0.25** | 0.35** |  |
|  | **ST_VV_DIS** |  |  |  |  |  |  |  |  |  |  |  | 1 | -0.48** | 0.39** | 0.35** |  |
|  | **ST_VV_COR** |  |  |  |  |  |  |  |  |  |  | 1 | -0.62** | 0.44** | 0.09 | 0.06 |  |
|  | **ST_VV_CON** |  |  |  |  |  |  |  |  |  | 1 | -0.61** | 0.95** | -0.32** | 0.37** | 0.37** |  |
|  | **ST_VV_ASM** |  |  |  |  |  |  |  |  | 1 | -0.11* | 0.24** | -0.22** | 0.86** | 0.50** | 0.59** |  |
|  | **ST_VH_MEA** |  |  |  |  |  |  |  | 1 | 0.63** | 0.20** | 0.13* | 0.18** | 0.42** | 0.82** | 0.84** |  |
|  | **ST_VH_HOM** |  |  |  |  |  |  | 1 | -0.55** | -0.13* | -0.53** | 0.41** | -0.60** | 0.18** | -0.57** | -0.55** |  |
|  | **ST_VH_ENT** |  |  |  |  |  | 1 | -0.82** | 0.53** | 0.09 | 0.49** | -0.23** | 0.59** | -0.17** | 0.59** | 0.54** |  |
|  | **ST_VH_COR** |  |  |  |  | 1 | -0.12* | 0.38** | 0.07 | 0.01 | -0.44** | 0.69** | -0.41** | 0.17** | 0.09 | 0.03 |  |
|  | **ST_VH_CON** |  |  |  | 1 | -0.36** | 0.47** | -0.57** | 0.63** | 0.32** | 0.57** | -0.22** | 0.49** | 0.16** | 0.51** | 0.55** |  |
|  | **ST_VH_ASM** |  |  | 1 | -0.34** | 0.13* | -0.81** | 0.82** | -0.41** | -0.02 | -0.38** | 0.30** | -0.47** | 0.24** | -0.46** | -0.41** |  |
|  | **SB_VV** |  | 1 | -0.27** | 0.39** | 0.05 | 0.35** | -0.35** | 0.57** | 0.41** | 0.22** | 0.06 | 0.21** | 0.27** | 0.62** | 0.64** |  |
|  | **SB_VH** | 1 | 0.57** | -0.40** | 0.65** | 0.05 | 0.53** | -0.55** | 0.97** | 0.59** | 0.22** | 0.12* | 0.20** | 0.38** | 0.81** | 0.82** |  |
|  | **AGB** | 0.17** | 0.11* | -0.14** | 0.17** | -0.02 | 0.15** | -0.11* | 0.16** | 0.11* | 0.08 | -0.06 | 0.06 | 0.08 | 0.12* | 0.15** |  |
|  | **Variable Name** | SB_VH | SB_VV | ST_VH_ASM | ST_VH_CON | ST_VH_COR | ST_VH_ENT | ST_VH_HOM | ST_VH_MEA | ST_VV_ASM | ST_VV_CON | ST_VV_COR | ST_VV_DIS | ST_VV_HOM | ST_VV_MEA | ST_VV_VAR |  |

1. Co-Innovation Center for Sustainable Forestry in Southern China, College of Forestry, Nanjing Forestry University, 210037, China. ^2^College of Forestry, Shanxi Agricultural University, Jinzhong, 030801, China. Correspondence and requests for materials should be addressed to M.L. (email: [lmy196727@njfu.edu.cn](mailto:lmy196727@njfu.edu.cn)) [↑](#footnote-ref-1)
